# Knowledge and use of antibiotics among health care users in Institute of public health of Belgrade

**DOI:** 10.1093/eurpub/ckac131.004

**Published:** 2022-10-25

**Authors:** G Belamaric, D Matijevic, D Vukovic, Z Bukumiric, S Mladenovic Jankovic, G Markovic, G Tamburkovski, K Vojvodic, M Jakovljevic

**Affiliations:** 1 Institute of Public Health of Belgrade, Belgrade, Serbia; 2 Institut of Social Medicine, Faculty of Medicine, Belgrade, Serbia; 3 Institute for Medical Statistics and Informatics, Faculty of Medicine University of Belgrade, Belgrade, Serbia; 4 Primary Health Center Zemun, Belgrade, Serbia

## Abstract

**Background:**

Antimicrobial resistance (AMR) is one of the major global public health threats that may lead to severe illness, hospital admissions, treatment failure and increasing of the health care costs. In order to address those challenges, the aims of this study were to examine the antibiotics consumption among the population of health care consumers in the Institute of Public Health of Belgrade, and their knowledge and attitudes regarding antibiotics compared to the inhabitants of EU and Japan.

**Methods:**

A cross-sectional study was conducted on a sample of 321 respondents who visited the Institute of Public Health of Belgrade in July 2021. The basic survey instrument was a Eurobarometer questionnaire (with the permission of the Directorate General for Communication European Commission). The obtained data were analyzed by methods of descriptive statistics, which included frequency distribution with percentages. In addition, the Chi-square test was used to examine the difference in frequencies.

**Results:**

More than half of all respondents used antibiotics in the previous 12 months (56.6%), majority of them with a doctor's prescription and 57.1% did some pre-testing (blood or urine test, swab) before or at the same time as using antibiotics. They used antibiotics most often for urinary infections (9,3%) and common cold (8,1%). We discovered that the knowledge of our respondents is somewhere in between comparing to the knowledge of the people from EU and Japan. 37.4% of our respondents knew that antibiotics are ineffective against cold and flu; 68.9% knew that unnecessary use of antibiotics makes them ineffective; 55.9% knew that taking antibiotics often leads to side effects such as diarrhea and 50.7% knew that antibiotics don't kill viruses.

**Conclusions:**

Knowledge about the antibiotics is insufficient and interventions of education, better informing and awareness of general public are necessary to encourage rational use of antibiotics.

**Key messages:**

• It is important to take evidence based interventions to reduce unnecessary use of antibiotics.

• This is the first study about antibiotics in Serbia which used the Eurobarometer model of research.

